# Antibiotic resistance plasmid composition and architecture in *Escherichia coli* isolates from meat

**DOI:** 10.1038/s41598-021-81683-w

**Published:** 2021-01-22

**Authors:** Tania S. Darphorn, Keshia Bel, Belinda B. Koenders-van Sint Anneland, Stanley Brul, Benno H. Ter Kuile

**Affiliations:** 1 Laboratory for Molecular Biology and Microbial Food Safety, Swammerdam Institute for Life Sciences, University of Amsterdam, Science Park 904, 1098 XH Amsterdam, The Netherlands; 2 Present Address: Wageningen Food Safety Research, Wageningen University and Research, Postbus 230, 6700 AE Wageningen, The Netherlands; 3 Netherlands Food and Consumer Product Safety Authority, Office for Risk Assessment, Utrecht, The Netherlands

**Keywords:** Microbiology, Antimicrobials, Antimicrobial resistance

## Abstract

Resistance plasmids play a crucial role in the transfer of antimicrobial resistance from the veterinary sector to human healthcare. In this study plasmids from foodborne *Escherichia coli* isolates with a known (ES)BL or tetracycline resistance were sequenced entirely with short- and long-read technologies to obtain insight into their composition and to identify driving factors for spreading. Resistant foodborne *E. coli* isolates often contained several plasmids coding for resistance to various antimicrobials. Most plasmids were large and contained multiple resistance genes in addition to the selected resistance gene. The majority of plasmids belonged to the IncI, IncF and IncX incompatibility groups. Conserved and variable regions could be distinguished in each of the plasmid groups. Clusters containing resistance genes were located in the variable regions. Tetracycline and (extended spectrum) beta-lactamase resistance genes were each situated in separate clusters, but sulphonamide, macrolide and aminoglycoside formed one cluster and lincosamide and aminoglycoside another. In most plasmids, addiction systems were found to maintain presence in the cell.

## Introduction

Resistance plasmids are instrumental in spreading resistance within and between veterinary and human healthcare^1–4^. The spread of resistance genes happens fast between cells of similar, but also different species of bacteria^5,6^. With increasing frequency *Escherichia coli* infections become untreatable due to extended spectrum beta-lactamases (ESBL) or beta-lactamases (BL) encoded on resistance plasmids and the WHO assigned highest priority to research on this subject^7^. In livestock (ES)BL and tetracycline resistance, which are mostly plasmid mediated, cause the greatest risks^8^. Tetracycline resistance was present in 77% of *E. coli* strains isolated from a swine feeding facility^9^ and chicken meat isolates in Germany showed high prevalences of (ES)BLs with the highest resistance percentage found for cefotaxime (74%)^10^. This antibiotic resistance can transfer from cattle directly to farmers^11,12^ as well as from foodstuffs to human healthcare^13^. Sources such as uncooked meat can spread antibiotic resistance^14^. To better understand the spreading of resistance mediated by plasmids, we need to understand the composition and maintenance systems of these plasmids.

Plasmids are classified usually by incompatibility group^15^. Incompatibility is assumed to be caused by competition for replication systems^16^. The idea is that two plasmids that compete for the same replication system cannot co-exist within a cell line over multiple generations. This characteristic is used to classify plasmids accordingly. The most common incompatibility group associated with (ES)BL resistance is IncF^17^. (ES)BL resistance has also been found in other incompatibility groups that are considered endemic resistance plasmids in *Enterobacteriaceae* such as IncI.

Plasmids contain a conserved region and a variable region^18^. The conserved region harbors the incompatibility group/replication genes, the genes for transfer and sometimes those for maintenance of the plasmid^19,20^. These genes are found to be similar per incompatibility group. The variable region contains mostly accessory genes such as resistance genes and can differ from plasmid to plasmid^18^. Transfer systems and mechanisms have been extensively described as well as some maintenance systems such as genes involved in partitioning^21–25^. Resistance genes have been researched in depth on function^26^. The composition of a single plasmid isolated from *Aeromonas hydrophila* was examined by comparative sequence analysis grouping the genes mainly based on function^27^. Comparison of this plasmid with sequences of similar plasmids in databases resulted in patterns found in similar plasmids from different species, including *E. coli*, and from different hosts such as human, bovine, beef, chicken and fish. The same prevalence of comparable plasmids was found using similar approaches across different species and a wide variety of locations^28,29^.

Analysis of the overall composition of resistance plasmids may provide insights into the spreading dynamics and what type of resistance plasmids can be transferred from livestock to human health care. Most studies have focused on certain elements within these plasmids, such as the transfer system^22,30^, resistance genes^11,17,31–34^, addiction system^25,35,36^, etc. While this proves useful information in their respective fields, the analysis of all information encoded on plasmids can discern patterns that explicate how plasmids function and behave. This can in turn explain the spread of antibiotic resistance mediated by these plasmids.

This research focusses on the composition and architecture of plasmids from veterinary *E. coli* species, given their role in spreading resistance to human health care. Two main questions are addressed: is it possible to distinguish specific regions or gene clusters in these plasmids that are linked to resistance, transfer, or maintenance? Are resistance gene regions or clusters conserved or variable within a plasmid and can this explain the mobility of the resistance genes? To answer these questions, plasmids present in *E. coli* isolated from chicken, turkey and beef that coded for (ES)BL or tetracycline resistance were sequenced using both long and short read technology. The results were hybrid assembled and analyzed for their composition and architecture. The entire plasmids per strain were mapped and analyzed for patterns. These patterns are used to explain epidemiological observations.

## Results

**Figure 1:**
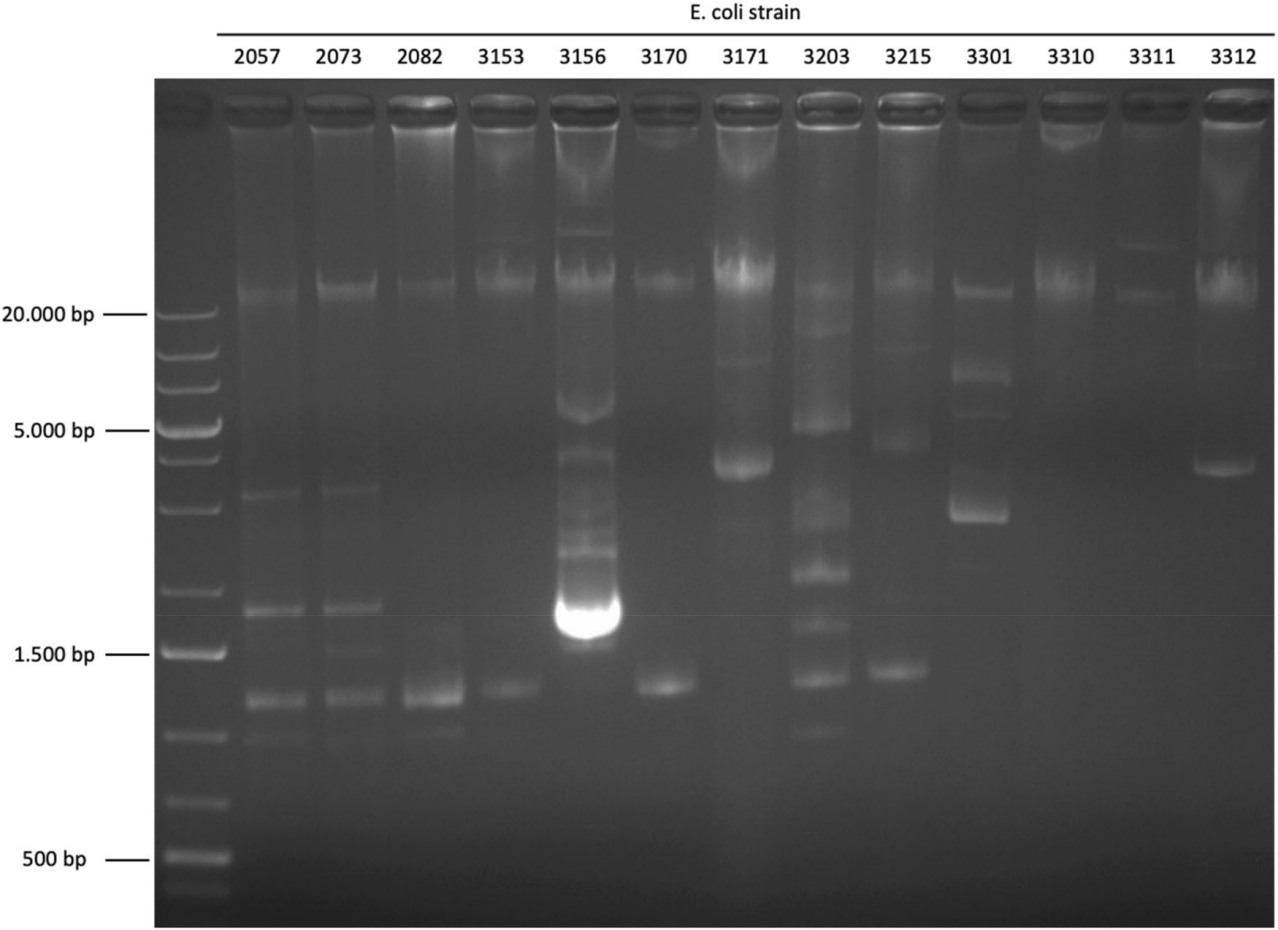
Gel photo showing different bands/plasmids isolated from different strains. Larger plasmids may have remained in or near the slots. Bands close in proximity of each other and with decreasing intensity suggest supercoiling.

Gel electrophoresis of plasmids isolated from different *E. coli* strains from meat destined for the consumer market shows that most strains contained several plasmids of different sizes (Fig. 1). Figure 1 should only be regarded as an illustration since the sizes are not determined well due to supercoiling and because the larger plasmids were underrepresented in these particular samples. Out of 17 strains analyzed 14 contained more than one plasmid (Table 1). Ample quantities of isolated plasmids were analyzed by next generation de novo-sequencing in order to obtain sufficient genetic material also of the largest plasmids, up to 190 kb.

**Table 1:** Plasmid/replicon types shown per *E. coli* strain with corresponding size, resistance genes and addiction systems.

*E. coli* strain	Plasmids; replicon type^a^	Size (bps)	Resistance genes detected in plasmid	Addiction system detected in plasmid
ESBL2057	IncI1	122,058	*aadA5, sul2, bla*_CTX-M-1_*, dfrA7*	*phd-doc*
	IncX4	38,612		*relE-relB/stbD, hicAB*
ESBL2073	IncI1	114,036	*bla*_CTX-M-1_*, aadA5, dfrA17, sul2*	*phd-doc*
	IncX4	38,586		*relE-relB/stbD, hicAB*
ESBL2082	IncI1-1	120,106	*aadA, aadA, cmlA1, aadA, sul3, bla*_SHV-12_*, tetAR, bla*_TEM-1B_	*phd-doc*
	IncX4	44,059	*lnuG(orB), aadA, bla*_TEM_	*hicAB*
	IncX1	42,080	*tetAR, bla*_TEM-1B_	
	IncFIB/FII	122,058	*macAB*	*phd-doc, hok*
	IncFIB(phage)	110,085		
ESBL3153	IncI1	101,623	*bla*_CTX-M-1_*, dfrA17, aadA5, sul2*	*phd-doc*
	IncX4	31,172		*hicAB*
	IncFII	67,397		*hok-gef, higAB*
ESBL3156	IncB/O/K/Z	89,311	*bla*_CMY-2_	*relE-relB/stbD, hok-gef*
	IncX4	44,183	*lnuG(orB), aadA24, bla*_TEM-1B_	*hicAB*
	IncFIB/FII	104,756		*phd-doc*
ESBL3171	IncI1	93,069	*bla*_CMY-2_	*phd-doc, ccdAB*
	IncFII	129,972		*vapBC, ccdAB, relE-relB/stbD, hok-gef*
ESBL3203	unknown IncI-like	105,181	*dfrA12, cmlA1, sul3, aadA1, bla*_CTX-M-8_	*doc*
	IncFII	–	*dfrA2, aadA1, sul2*	*hok-gef*
ESBL3215	IncI2	–	*bla*_TEM-52c_	*relE-relB/stbD, VapBC*
	IncX4	33,266	*mcr-1.1*	*hicAB*
	IncFIB/FIC	94,729	*macA*	*vapBC, relE-relB/stbD, hok-gef*
	phage plasmid	49,269		
ESBL3227	IncI1-I	116,368	*sul2, bla*_CTX-M-2_*, sul2, aac3-*Via*, aadA1*	*phd-doc*
	IncFIB/FII/Q1	149,552	*sul2, aph3′'-Ib (2x), aph6-Id, dfrA1, ant(3′'), sul2, tetRA, catA, bla*_TEM-1b_	*phd-doc, hok-gef, vapBC*
ESBL3231	IncN	44,460	*aadA1, dfrA1, bla*_SHV-12_*, bla*_TEM_	
	IncFIB/FII	142,299	*aadA15, aadA24, qnrS1, lnuFG, sul3, tetAR*	*phd-doc*
	P0111	92,432		
ESBL3277	IncFIB/FIC	132,452	*dfrA14, tetAR, bla*_CTX-M-55_*, aac3-Iia, aadA1(2x), sul3, floR*	*pemKI, hok-gef, vapBC, sok*
ESBL3284	IncFIB/FII	109,303	*macAB*	
ESBL3288	IncFIA/FIB/FII	126,935	*dfrA17, sul1, bla*_CTX-M-15_*, aac3-Iia, aadA5*	*hok-gef, pemKI, vapBC, ccdAB*
ESBL3301	Inc1	104,138	*bla*_CTX-M-1_	*relE-relB/stbD*
	IncX1	58,179	*tetAR*	*doc*
	IncFIB/FII	105,961		*hok-gef, vapBC, doc, ccdAB*
ESBL3310	IncB/O/K/Z	119,594	*sul1, bla*_CMY-2_*, tetAR, aac3-*VIa*, aadA1*	*phd-doc*
	IncFIB/FIC	192,264		*vapBC, ccdAB, hok-gef, relE-relB/stbD, phd-doc*
ESBL3311	IncX1	41,269	*floR*	*relE-relB/stbD*
	incFIB	38,494	*bla*_TEM-1A (2x)_*, tetAR*	
	IncR	49,742	*bla*_CTX_M-1_	
ESBL3312	IncX1	41,210	*floR*	*relE-relB/stbD*
	incFIB	38,446	*bla*_TEM-1A (2x)_*, tetAR*	
	IncR	50,101	*bla*_CTX_M-1_	

Missing plasmid sizes are due imperfect assemblies that make a correct estimation of the plasmid impossible.

^a^Multiple replicon types in one row means a plasmid contains all these replicons.

None of the strains contained two plasmids belonging to the same incompatibility group (Table1). The most common incompatibility groups in this set of strains are part of the IncI-family, IncX-family and IncF-family 34 out of 42 plasmids. IncI was found far more in poultry isolates (eight out of nine plasmids) than in beef isolates (one out of nine plasmids). All other plasmid types were distributed equally over the different kinds of meat. Other plasmids found were IncB/O/K/Z, IncR, IncN and P0111 as well as some plasmids containing mainly phage like genes (phage/phage plasmid) (8 out of 42 plasmids). Out of 16 plasmids containing IncF-group replicons, ten contained two or three replicons within a single plasmid. While this is not common for other incompatibility groups, multiple replicons found in a plasmid is common in the IncF-family^37^. The size of plasmids corresponded to the replicon-type for IncI and IncX, at between 90 and 120 kb and 30–60 kb, respectively. The plasmids containing IncF replicons differed in size: from around 40 kb up to more than 190 kb. Measurements of the minimal inhibitory concentration (MIC) using six antimicrobials of five different classes revealed that all strains were resistant to more than one antibiotic (data not shown). The MIC showed in some cases discrepancies with the AMR genes found. This was in most cases due to a resistance that is most likely genomic, since it showed up in the MIC but not as AMR gene on the plasmid. The most striking difference was found for strain ESBL3284, were it was expected to find beta-lactam gene CMY-2 on a plasmid (as screened before by WBVR) and showed a slightly higher MIC for ampicillin and amoxicillin than sensitive strains. The plasmid however did not harbour the CMY-2 gene and hence this is most likely a genomic resistance. Sequencing of the genome has not been performed, but transfer experiment data suggest that since no successful transfers were found, this gene might be genomic. Out of 42 plasmids recovered, 31 contained between 1 and 10 resistance genes (Table 1). Most plasmids coded for multiple resistant genes (18 out of 42), some did not contain resistance genes (13 out of 42) and 13 contained only a single resistance gene. The most common belonged to the (ES)BL-group (*bla*), others were part of the tetracycline group (*tet*), aminoglycoside group (*aad, aac, ant & aph),* sulphonamides (*sul*), trimethoprim (*dfr*), chloramphenicol (*cat*, *cml & flo*), fluoroquinolone (*qnr*), macrolides (*mac*), lincosamides (*lnu*) and colistin (*mcr*). The *macAB* and *floR* genes were never accompanied by another resistance gene on the same plasmid. Most plasmids contained a toxin-antitoxin system, also referred to as an addiction system^25^. Only 8 out of the 42 plasmids examined here did not contain such a system. The *phd-doc* system was mostly found in IncI replicon types, while *hicAB* or *relE-relB/stbD* were mostly associated with IncX replicon types. IncF-type plasmids contained a wide variety in addiction systems and often multiple systems were discovered in a single plasmid. In all, 16 plasmids contained only one addiction system, while the maximum number of addiction systems found was five.

**Figure 2:**
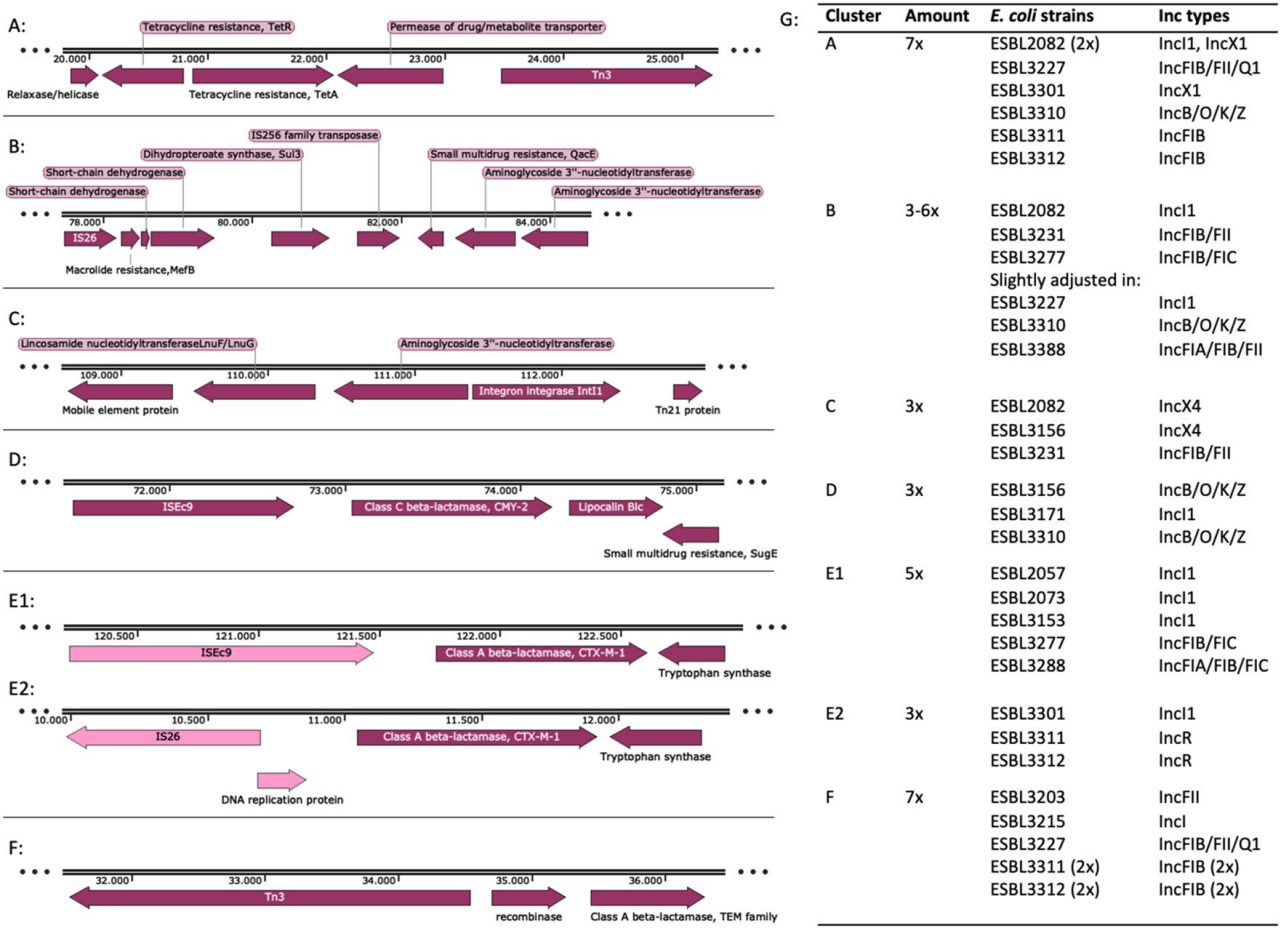
Shows six different resistance gene clusters found over different plasmids. Showing cluster (**A**) including genes coding for tetracycline resistance, cluster (**B**) with genes encoding for macrolide, sulphonamide and aminoglycoside resistance, and small multidrug resistance sugE, cluster (**C**) containing genes encoding for lincosamide and aminoglycoside resistance, cluster (**D**) containing specific beta-lactamase resistance gene blaCMY-2, cluster (**E**) containing specific beta-lactamase resistance gene CTX-M-1 in 2 versions and cluster (**F**) including specific beta-lactamase resistance gene from the TEM family. (**G**) contains a table showing the number of times a cluster was found in the dataset and the corresponding *E. coli* strain and Inc plasmid type.

The structures of the plasmids were analyzed for common elements and recurring gene clusters. The clusters described were selected on the basis of common elements in resistance genes. In Fig. 2 clusters are shown that were found in plasmids coding for beta-lactam, tetracycline, macrolide, sulphonamide and aminoglycoside resistance. Specific clusters encoding for beta-lactam resistance genes were identified including *bla*_CMY-*2*_, *bla*_CTX-M-1_ and genes from the *bla*_TEM_ family. The resistance gene clusters were accompanied by mobile genetic elements, such as transposases and insertion sequences. Some of the clusters contained an additional small multi-drug resistance gene. The tetracycline resistance cluster contained *tetA* and *tetR* next to a permease of the drug/metabolite transporter superfamily, a transposase from the *Tn3*-family and a relaxase (Fig. 2A). The macrolide/sulphonamide/aminoglycoside resistance cluster contained the mobile genetic element *IS26*, macrolide resistance gene *mefB*, sulphonamide resistance gene *sul3* (dihydropteroate synthase), a transposase, small multidrug resistance gene *qacE*, and 2 aminoglycoside resistance genes from the *aadA* family (Fig. 2B). Lincosamide/aminoglycoside cluster was associated with mobile genetic element *IL-IS_2,* integrase *intI1* and transposase *Tn21* (Fig. 2C)*.* The cluster around beta-lactamase *bla*_CMY-2_ included mobile genetic element *ISEc9*, lipocalin *blc*, small multidrug resistance gene *sugE* (Fig. 2D)*.* The cluster containing *bla*_CTX-M-1_ existed in two different modes: while always associated with a tryptophan synthase, it was either found next to insertion sequence *ISEc9* (Fig. 2E1) or next to a replication protein and insertion sequence *IS26* (Fig. 2E2). The plasmids described also contained other types of *bla*_CTX-M_ genes, but these where not located in the same cluster. Beta-lactamases of the *bla*_TEM_-family were often found in a cluster next to transposase *Tn3* and a recombinase (Fig. 2F). While the cluster around *bla*_CMY-2_ was always linked with the IncI replicon, the other clusters were distributed among different plasmid types. Additional clusters associated with resistance against heavy metals were also identified but are not described here.

Table 2 shows the specific subtypes found for the three most commonly found incompatibility groups in this data set: IncF, IncI and IncX. The IncF plasmids show the highest number of subtypes and only two of the subtypes are found twice. Some of the subtypes also include novel alleles not found in the database. The IncI plasmids show more similarities in subtypes, where subtype 3 and 12 are found multiple times. The IncX plasmids in this study can be split into two groups, since only the X1 and X4 subtypes were detected.

**Table 2:** Subtypes found per strain for the three most commonly found incompatibility groups: IncF, IncI and IncX.

*E. coli* strain	Subtypes found for:		
	IncF	IncI	IncX
ESBL2057		3	X4
ESBL2073		3	X4
ESBL2082		95	X1, X4
ESBL3153	F104:A−:B−	3	X4
ESBL3156	F4:A−:B1		X4
ESBL3171	F2:A−:B−^a^	12	
ESBL3203	F16:A−:B24	114	
ESBL3215	F89:A−:B53	Unique	X4
ESBL3227	F24:A−:B1	12	
ESBL3231	F24:A−:B1		
ESBL3277	F18:A−:B1		
ESBL3284	F−^a^:A−:B1		
ESBL3288	F31:A4:B1		
ESBL3301	F56:A−:B16	26	X1
ESBL3310	F18:A−:B20		
ESBL3311	F−:A−:B20		X1
ESBL3312	F−:A−:B20		X1

^a^Subtype contains a novel allele and can therefore not be fully typed.

**Figure 3:**
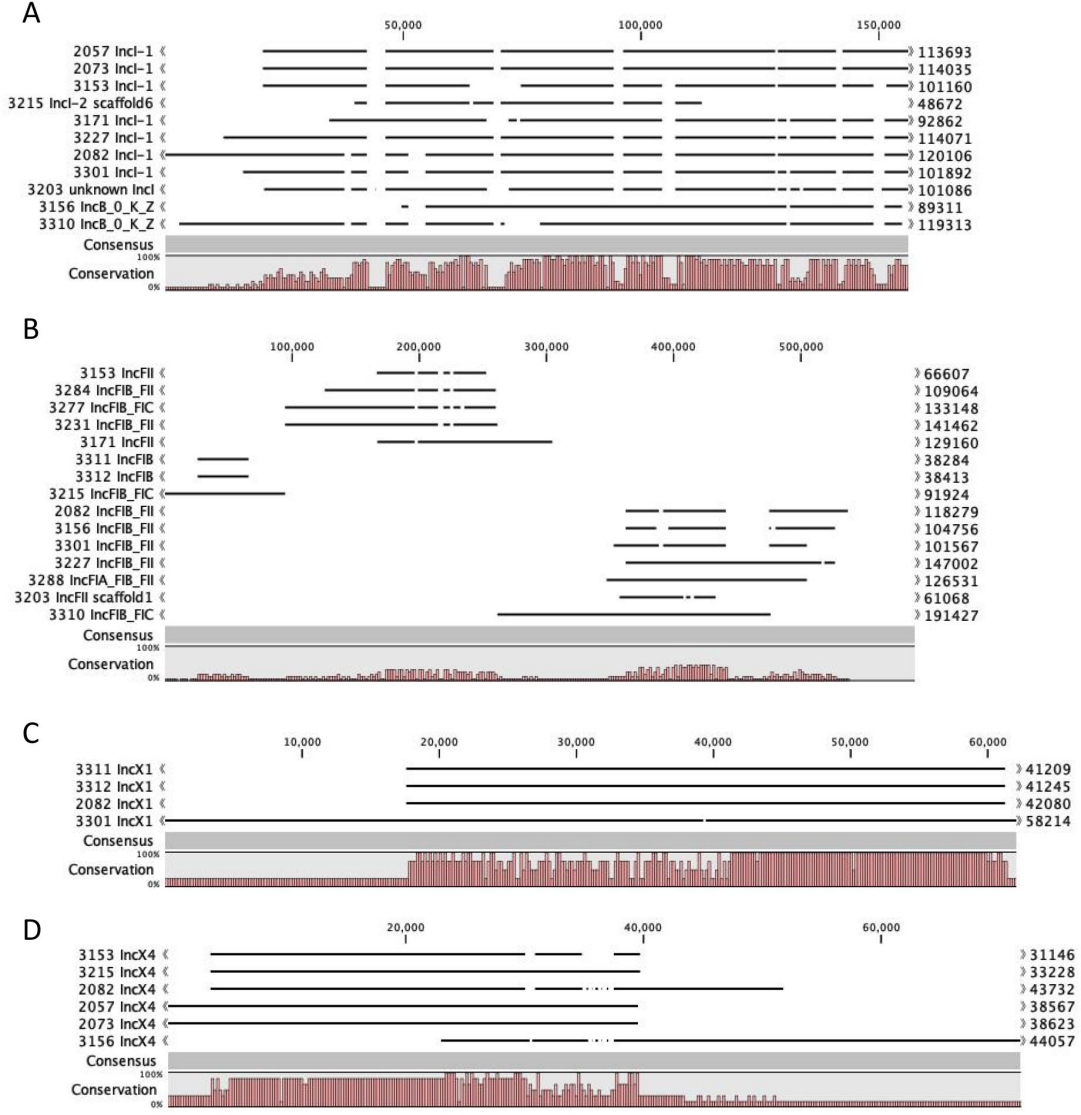
alignments showing the similarity between the same plasmid types: IncI-like (**A**), IncF-like (**B**), IncX1-like (**C**) and IncX4-like (**D**), within different *E. coli* strains. Imperfect assemblies are distinguished by scaffold in the name.

Figure 3 shows the homology of the four most commonly found plasmid/replicon types. The IncI containing plasmids showed the most overlap and least and smallest gaps (Fig. 3A). Subtype 3 as found in ESBL2057, 2073 and 3153 shows an even higher overlap, but in general the subtypes are all quite similar. The IncB/O/K/Z plasmids were also compared with the IncI plasmids, since they showed a high homology in architecture. IncF containing plasmids have the least amount of overlap. They can be grouped together in three sets of plasmids showing higher homology among each other. The subtypes do not seem to be linked to these three groups as well as the size of the plasmids. The IncX subtypes IncX1 and IncX4 were split into two groups which have high homology within, but not between each other. Both showed a high amount of conservation within their subtypes with few gaps. The clusters shown in Fig. 2 are located in these gaps. These gaps further contain accessory genes like, heavy metal or other types of resistances and addiction systems.

**Figure 4:**
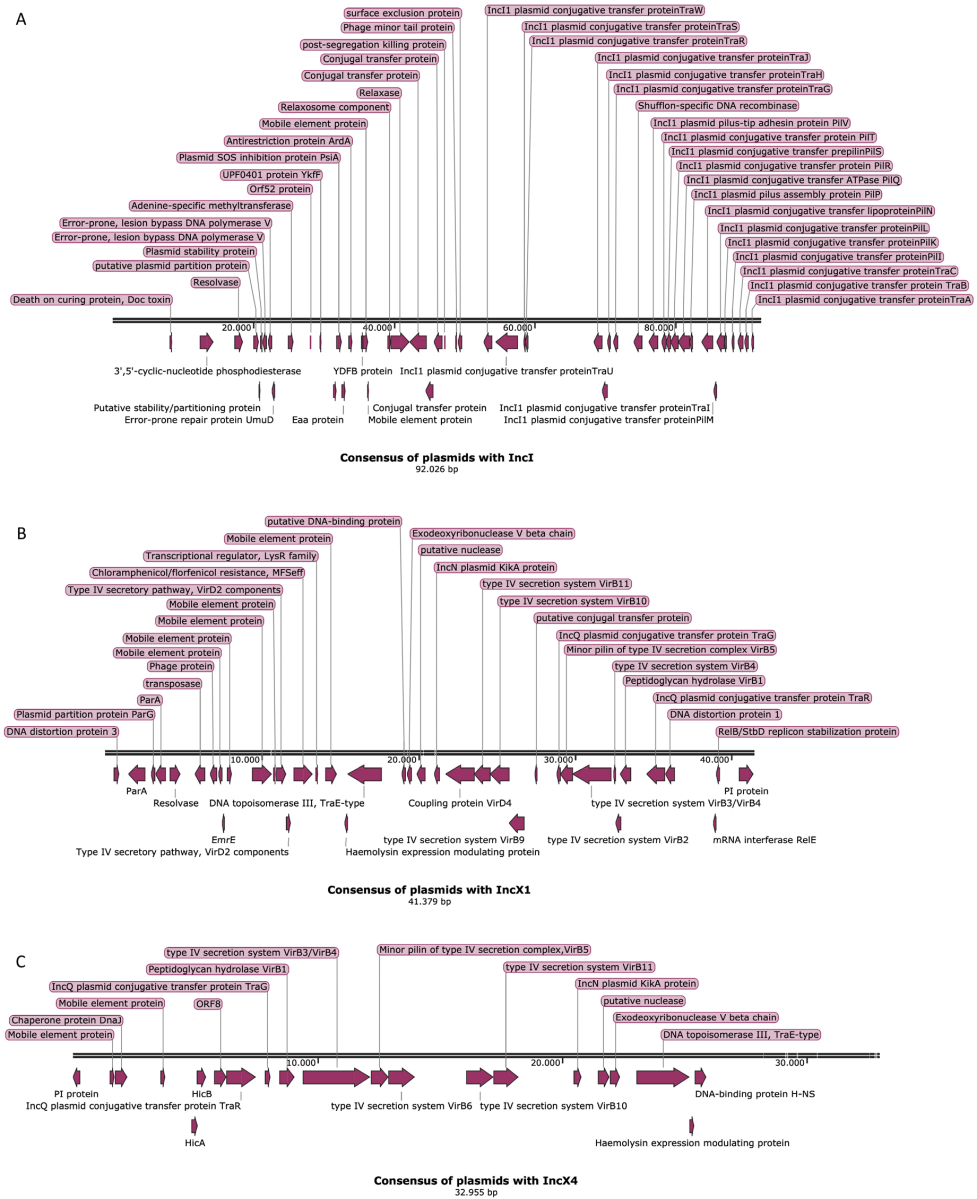
Annotated consensus sequences of the IncI (**A**), IncX1 (**B**) and IncX4 (**C**) type plasmids.

In Fig. 4, a single consensus sequence is presented for IncI, IncX1 and IncX4. Hypothetical proteins of unknown function have been omitted. An IncF consensus sequence could not be reconstructed, because of lower homology and higher variation between these plasmids. Regions can be identified in all consensus sequences that correspond to clusters of genes involved in plasmid transfer and clusters involved in plasmid maintenance. The IncI replicon containing plasmids all harbor plasmid transfer genes from the *tra*-family, where the IncX plasmids contain type IV secretion system genes (*virB*) for transfer. Plasmid maintenance genes that are preserved for the IncI plasmids contain the addiction system *phd-doc*, SOS inhibition genes (*psiAB*), some plasmid partition and stability genes, a surface exclusion gene and a post-segregation killing gene (unidentified addiction system). The IncX1 plasmids contained more plasmid maintenance genes than the IncX4 harboring plasmids, such as partition genes *parA* and *parG*, the *kikA* gene as well as the addiction system *relE*-*relB/stbD*. Interestingly, the IncX1 also included a preserved resistance gene for chloramphenicol, where the other two types of plasmids do not. The IncX4 plasmids had only one addiction system (*hicAB*) and the *kikA* gene as homologous genes involved in plasmid maintenance. Both IncX4 and IncX1 plasmids also had the replication gene conserved (PI protein) in contrast to IncI plasmids which contained multiple subtypes of the different IncI-family replication genes. Some mobile elements were also preserved within the consensus sequences. The two separate IncX consensus sequences had a few of the same genes in common, especially belonging the type IV secretion system.

**Figure 5:**
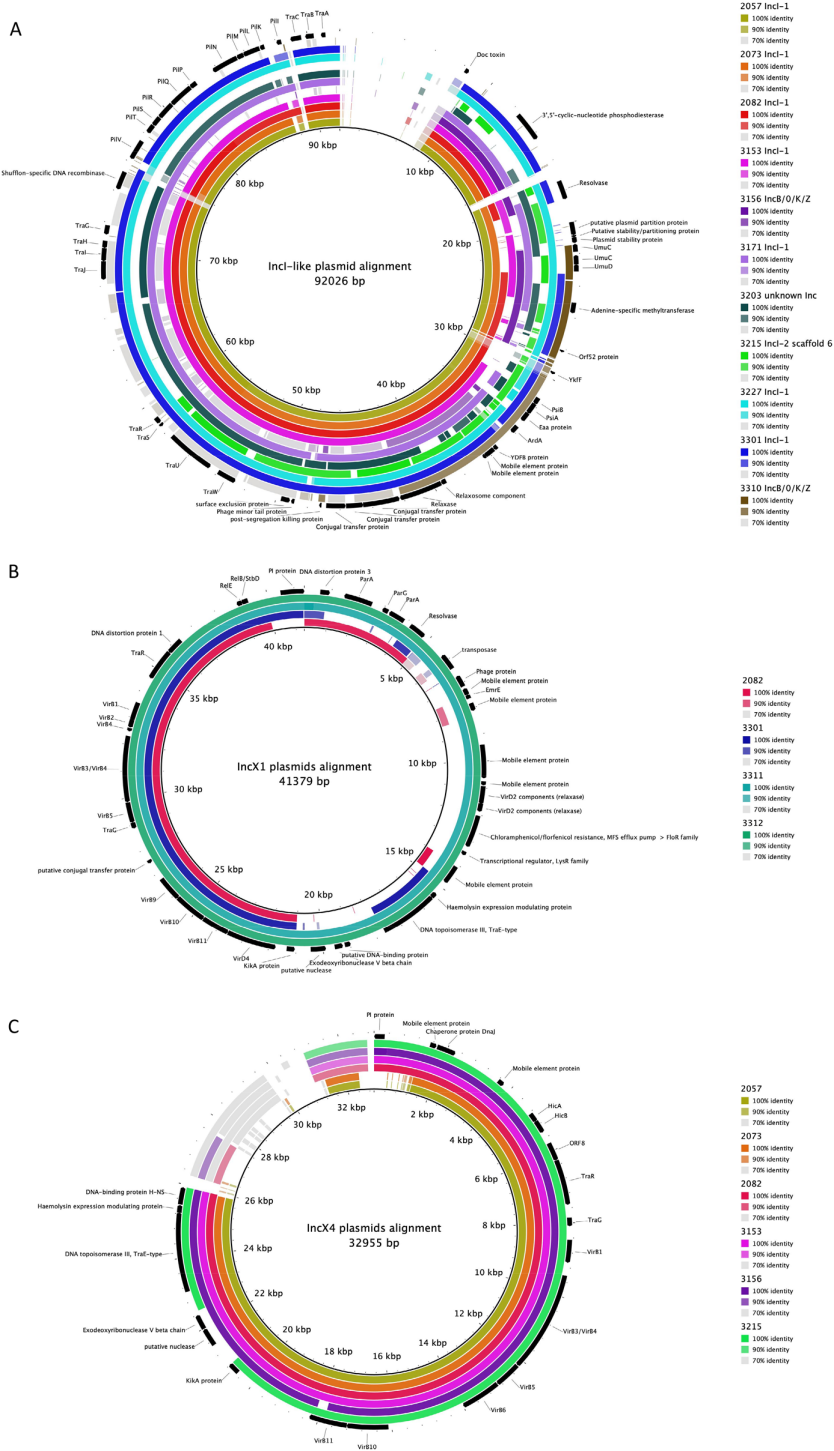
Alignment of IncI plasmids (**A**), IncX1 plasmids (**B**) and IncX4 plasmids (**C**) showing the alignment of all similar plasmids among all *E. coli* strains tested in this dataset against the annotated consensus sequence. Colors indicate the homology, where full color show 100% identity, slightly lighter color indicates 90% identity or higher, grey indicates 70% identity or higher and no color indicates less than 70% homology.

Three examples of plasmid architecture based on consensus domains are presented in Fig. 5. The replicons and sequences of IncI immediately downstream from the origin were considerably different from each other, causing an area with little alignment, defined as less than 70% homology (Fig. 5A). The remainder of the plasmids showed increasing homology as demonstrated by the colors and the decreasing number of gaps. Five clusters can be identified. A first cluster appears after between 9 and 17 kb and includes the *doc* toxin and 3′,5′-cyclic-nucleotide phosphodiesterase. Starting around 20–30 kb we find a cluster including a resolvase, putative plasmid partition protein, plasmid stability proteins, lesion bypass DNA polymerase *umuCD* and adenine specific methyltransferases. The third cluster spans from around 30 to 76 kb containing *ykfF, psiAB, eea, ardA, ydfB*, mobile element proteins, relaxosome component, relaxase, conjugal transfer proteins, post-segregation killing protein, phage minor tail protein, surface exclusion protein, transfer proteins *traW, traU, traS, traR, traJ, traI, traH, traG* and a shufflon-specific DNA recombinase. From 76 to 88 kb, a fourth cluster is described containing only pilus or conjugative transfer proteins known as *pilV, pilT, pilS, pilR, pilQ, pilP, pilN, pilM, pill, pilK* and *pilI*. The last cluster contains three more transfer proteins*, traA, traB* and *traC* and spans over the section from 88 to 91 kb.

IncX1 has higher homology and shows no separated clusters, but instead consists of one complete plasmid (Fig. 5B). The plasmid starts with a DNA distortion protein, followed by partition protein *parA, parG* and again *parA*, then a section containing mobile elements proteins including resolvase, transposase and relaxases, followed by a chloramphenicol resistance gene (*floR*), transcriptional regulator *lysR*, haemolysin expression modulating protein, transfer gene of the *traE*-type, *kikA*, and a cascade of type IV secretion system genes, *virD4, virB11, virB10, virB9, traG, virB5, virB3, virB4, virB2, virB1* and *traR*. The last bit includes another DNA distortion protein, addiction system *relE-relB/stbD* and ends with the replication protein (PI protein).

The IncX4 alignment can be split into two parts: one of high homology that contains all known genes and one with lower homology where no known genes could be annotated in the tail of the plasmid (Fig. 5C). The highly homologous part starts with the replication protein (PI protein), followed by mobile element proteins, *dnaJ*, addiction system *hicAB*, transfer genes belonging to the type IV secretion system including *traR, traG, virB1, virB3/4, virB5, virB6, virB10* and *virB11*. Furthermore, it also contains the *kikA* protein, a putative nuclease, a *traR*-type gene, haemolysin expression modulating protein and a DNA-binding protein.

## Discussion

Most research on resistance plasmids has focused either on comparisons of resistance genes, or on one type of plasmid, or on mobile genetic elements^38^, as opposed to overall plasmid composition in multiple strains. In this study, we attempt to find common elements and unifying principles in sets of plasmids found in *E. coli* resistant to antimicrobials. While in no way repudiating the value of other approaches, we feel that this point of view focusing on architecture and shared components adds novel insights to the understanding of plasmids.

Two separate types of regions can be distinguished in plasmids; conserved and variable regions^18^. The conserved regions mostly contain genes involved in replication, stability and mobility, while the variable regions contain accessory genes. In the plasmids described in this study, the consensus sequence, defined as the conserved regions per incompatibility group, is composed of genes for plasmid transfer and maintenance. The variable regions that contain the resistance genes are located in the gaps seen in the consensus sequence or in mobile genetic element sites (Figs. 2, 4 and 5). This implies that the plasmid can preserve its integrity by inserting new genes solely into designated regions and between essential genes.

Within these *E. coli* strains the IncI plasmids were more conserved compared to IncF-like plasmids. A variation in conserved regions within the IncF group was earlier also observed by Fernandez-Lopez et al.^19^, separating the IncF group into five different classes. These classes, however, did not always correspond to the range found in this study. The consensus of IncI, IncX1 and IncX4 outlines the minimum requirement for a plasmid to be classified as this type of plasmid according to this dataset. This set of similar genes suggests that crucial genes for classification are those that are involved in plasmid maintenance and plasmid transfer^18,39^. Possibly these represent the minimum number of genes necessary for transfer and maintenance for the corresponding plasmid type.

This study supports the theory of incompatibility^16^, as no two plasmids belonging to the same incompatibility group were located in the same strain. Incompatibility is caused by the competition between two plasmids using the same replication and partitioning system^40,41^. Hence, in theory, plasmids of two similar but not identical incompatibility groups could co-exist within one strain, if these plasmids also harbor other genes enhancing their survival such as partitioning genes or addiction systems. The less similar their incompatibility region is, the more likely plasmids are to be able to coexist within one cell^42^. In contrast, it is common to find multiple replicons of the same incompatibility group within one plasmid, especially for IncF and IncI type plasmids^43^.

Strains to be included in the study were selected for either (ES)BL or tetracycline resistance, not for multiple resistance, though further characterization often showed this. Resistance genes were frequently assembled in clusters flanked by MGEs suggesting that multiple types of resistance can be easily transferred from plasmid to plasmid or from plasmid to chromosome^38^. Since clusters of this kind are highly mobile, they stimulate the spread of antimicrobial resistance^44^. The presence of these clusters sometimes multiple times within a single strain is in line with the concept of mobile genetic elements being able to move around separately from the plasmid^38,45^.

Two types of multiple resistance clusters have been identified: (1) Sulphonamide, macrolide and aminoglycoside resistance and (2) lincosamide and aminoglycoside resistance (Fig. 2). The (extended spectrum) beta-lactamases and tetracycline resistance genes are more often located each in a separate cluster, suggesting that these MGEs only transfer one type of resistance. In a dataset comprising 2522 bacterial genomes antimicrobial resistance clusters were positioned close to heavy metal or chemical resistance genes^46^. This observation supports the idea that these categories of genes only insert within specific places within a plasmid rather than randomly^47^. Tetracycline clusters flanked by *Tn10* are wide-spread in gram-negative and gram-positive bacteria^48,49^.

Beta-lactam resistance “clusters” are associated mostly with the same IS elements or transposases but not necessarily always with the same “accessory genes”^31,45,48–52^. The various clusters of *CTX-M* genes are highly prevalent in plasmids^32,53^. This in turn suggests that this class of genes might be transferred more often from cell to cell or from plasmid to plasmid than other resistance genes. Indeed, other studies have shown that ESBL plasmids can be transferred at a higher rate than tetracycline resistance plasmids^54,55^. The presence of *TEM* clusters in multiple plasmids within one cell suggests that the transposition of these genes had already taken place. The *bla*_CMY-2_ cluster is associated in this dataset only to IncI-like plasmids, as opposed to other findings that the *bla*_CMY-2_ gene itself is also associated to other replicon types such as IncA/C, IncFIA-FIB, IncNT^53^ as well as the entire cluster being found in IncK and/or IncB/O plasmids^51^. When beta-lactam resistance is induced, a section of the genome of *E. coli* that contains a similar cluster including *ampC,* the chromosomal equivalent of *bla*_CMY-2_, is replicated in high numbers^56^.

Addiction systems that allow plasmids to maintain themselves within a cell by producing a stable toxin and unstable anti-toxin^25^ are widespread^35,36^. Addiction systems can explain why multiple large plasmids remain in the cell over generations even in the absence of selective pressure^57^. Almost all plasmids in this study contained a toxin-antitoxin system. Whether these addiction systems are associated with certain plasmid types is a matter of debate. Zielenkiewicz and Ceglowski^25^ state: “there is no uniform pattern for the placement of stability cassettes on plasmids”. Addiction systems were judged to be randomly distributed over plasmids rather than to be associated with specific types^24^. Later a link was found between the beta-lactamase *bla*_CTX-M_ and the addiction genes *ccdAB, vagCD*, and *pndAC*^58^. In the present study the *phd-doc* system was located on the IncI plasmids and the *hicAB* and *relE-relB/stbD* systems on the IncX plasmids, also suggesting a correlation between addiction systems and incompatibility types.

In conclusion, resistance plasmids recovered from foodborne *E. coli* strains share common characteristics in structure and contents that correlate to their incompatibility groups. The shared features include essential genes for plasmid transfer and maintenance. Most plasmids maintain their presence in the cell with the assistance of addiction systems^59^. Specific resistance clusters can be positioned in any of the plasmids investigated. As a result, resistance genes move easily and rapidly between plasmids and from plasmid to chromosomes^5,6^.

## Material and methods

### Bacterial strains

*Escherichia coli* strains were isolated from foodstuffs by the Dutch Food and Consumer Product Safety Authority (NVWA) and characterized by Wageningen Bioveterinary Research (WBVR). Dr. Kees Veldman of WBVR selected and donated 28 strains that were known to contain plasmids. Strains originated from chicken, turkey or bovine meat and were selected for having either a beta-lactam or tetracycline resistance. The resistance was established by the NVWA by sensitivity tests^60^ and confirmed afterwards using MIC assays^61^. Plasmid presence was initially confirmed by the by detection of the incompatibility group^62^ and later confirmed by successful plasmid transfer experiments^54^ and gel electrophoresis. All *E. coli* strains were cultivated before plasmid isolation by first plating the strains on LB-agar plates with 64 μg/ml amoxicillin or tetracycline (to prevent plasmid loss) overnight at 37 degrees Celsius. One colony was picked and grown first in 5 ml LB with antibiotics before scaling up to 400 ml LB with antibiotics. From the final volume, cell pellets were collected.

### Plasmid preparation and sequencing

The plasmids were isolated using a Qiagen Plasmid Maxi Kit, checked for purity with Nanodrop and if samples had low concentration of DNA or were too contaminated with salts they were purified with ethanol precipitation. Sequencing was performed by BaseClear B.V. (Leiden, the Netherlands) using Illumina NovaSeq 6000 and PacBio systems. Hybrid de novo assembly was performed using both the generated Illumina short read and PacBio long read data. In most cases several plasmids originated from a single strain and as a result the correct assembly of the plasmids turned out to be problematic and to pose severe methodological problems. Short read data only were insufficient for plasmid assembly. In the end hybrid assembly combining long and short read data has provided high quality scaffolds that are reliable and translated back into single plasmids. Hybrid assembly was performed by first improving the quality of the Illumina reads by trimming of low-quality bases using BBDuk, part of BBMap suite version 36.77 (Bushnell B., sourceforge.net/projects/bbmap/). High-quality reads were assembled into contigs using ABySS version 2.0.2^63^. The long reads were mapped to the draft assembly using BLASR version 1.3.1^64^. Based on these alignments, the contigs were linked together and placed into scaffolds. The orientation, order, and distance between the contigs were estimated using SSPACE-LongRead version 1.0^65^. Using Illumina reads, gapped regions within scaffolds were (partially) closed using GapFiller version 1.10^66^. Finally, assembly errors and the nucleotide disagreements between the Illumina reads and scaffold sequences were corrected using Pilon version 1.21^67^. The assembly of strains that afterwards still showed too low quality were not included in this paper. Low quality refers to too many unassembled small scaffolds, too many gaps and too low coverage of reads mapping to a scaffold. Hybrid assembly can sometimes result in the artificial fusion of plasmids within one scaffold, as also seen by Margos et al.^68^. This artifact was mostly caused by identical regions present in two plasmids within the same cell. These were separated afterwards, based on gel photos showing multiple bands, Illumina only data showing these specific plasmids as separated and transfer experiments resulting in the transfer of single separated plasmids. Fusion plasmids or hybrid plasmids do also occur in nature and this needs to be taken into account before splitting fused plasmids^69^. The scaffolds thus obtained were tested for circularity to determine if one scaffold corresponds to one full plasmid. This was determined by considering whether the scaffolds contained a full sequence of a plasmid or an overlapping gene found partly at the start and partly at the end of the sequence, as checked through BLAST (http://www.ncbi.nlm.nih.gov/BLAST/). Circularity was also based on the annotation similarities between the start and end of the sequence, such as genes commonly found next to each other. Scaffolds not corresponding to a full sequence are distinguished from the other plasmids by name. Establishing circularity was also attempted using other softwares and then seems highly likely, but could not be proven beyond any doubt.

### Data analysis

The scaffold sequences were screened for resistance genes and their incompatibility group using CGEs ResFinder 4.0^70^ and PlasmidFinder 2.1^71^. Full annotation was performed afterwards using RAST 2.0^72^. The annotated sequences were further analyzed using Snapgene viewer 5.1.7 (from Insightful Science; available at snapgene.com). Plasmid subtyping was done using CGEs pMLST 2.0^71^. Plasmid alignment for the specific incompatibility groups was done using CLC Genomics Workbench 7 (https://digitalinsights.qiagen.com) and BRIG 0.95^73^. Imperfectly assembled plasmids were included in the alignment, but are distinguished by adding scaffold to the name. CLC workbench alignments provided a general view of matching parts in the sequences and were used to create a consensus sequence for each incompatibility group. BRIG was used to visualize the annotated consensus and the matching plasmids sequences.
